# Sulfur amino acid supplementation displays therapeutic potential in a *C. elegans* model of Duchenne muscular dystrophy

**DOI:** 10.1038/s42003-022-04212-z

**Published:** 2022-11-16

**Authors:** Rebecca A. Ellwood, Luke Slade, Jonathan Lewis, Roberta Torregrossa, Surabhi Sudevan, Mathew Piasecki, Matthew Whiteman, Timothy Etheridge, Nathaniel J. Szewczyk

**Affiliations:** 1 MRC Versus Arthritis Centre for Musculoskeletal Ageing Research, Royal Derby Hospital, University of Nottingham & National Institute for Health Research Nottingham Biomedical Research Centre, Derby, DE22 3DT UK; 2 Sport and Health Sciences, University of Exeter, St Luke’s Campus, Exeter, EX1 2LU UK; 3 University of Exeter Medical School, University of Exeter, St. Luke’s Campus, Exeter, EX1 2LU UK; 4 Ohio Musculoskeletal and Neurologic Institute and Department of Biomedical Sciences, Ohio University, Athens, OH 45701 USA

**Keywords:** Nutrition disorders, Physiology

## Abstract

Mutations in the dystrophin gene cause Duchenne muscular dystrophy (DMD), a common muscle disease that manifests with muscle weakness, wasting, and degeneration. An emerging theme in DMD pathophysiology is an intramuscular deficit in the gasotransmitter hydrogen sulfide (H_2_S). Here we show that the *C. elegans* DMD model displays reduced levels of H_2_S and expression of genes required for sulfur metabolism. These reductions can be offset by increasing bioavailability of sulfur containing amino acids (L-methionine, L-homocysteine, L-cysteine, L-glutathione, and L-taurine), augmenting healthspan primarily via improved calcium regulation, mitochondrial structure and delayed muscle cell death. Additionally, we show distinct differences in preservation mechanisms between sulfur amino acid vs H_2_S administration, despite similarities in required health-preserving pathways. Our results suggest that the H_2_S deficit in DMD is likely caused by altered sulfur metabolism and that modulation of this pathway may improve DMD muscle health via multiple evolutionarily conserved mechanisms.

## Abstract (web summary)

A *C. elegans* model of Duchenne muscular dystrophy reveals a potential role for disrupted sulfur metabolism in the disease and thus the therapeutic potential of sulfur amino acid supplementation.

## Introduction

Duchenne muscular dystrophy (DMD) is an X-linked recessive genetic disorder mainly associated with live male births^1^. It is well known that DMD is caused by mutations in the dystrophin gene, resulting in progressive muscle damage and degeneration due to destabilisation of the dystrophin-glycoprotein complex (DGC) (a large multicomponent complex responsible for mechanical stability)^2^. It is also known that the absence of dystrophin results in a loss of calcium homeostasis, mitochondrial dysfunction, and oxidative stress^3,4^. Effective therapeutics for DMD remain limited despite urgent, unmet clinical needs. The current gold standard treatment are glucocorticoids and more recently, three exon-skipping therapies have been approved by the Food and Drug Administration (FDA)^5–7^. Despite a slight improvement in life expectancy from these compounds, the average time of death is still late twenties, highlighting the need for alternative studies and treatments^8–10^.

Homologues of dystrophin have been identified in several different animals including mice, dogs, rats, and invertebrates^10^. Animal models have proven themselves important in the development of potential therapeutics, with high-throughput screening routine practise in Zebrafish and *Caenorhabditis elegans* DMD models. There are currently eleven known drug classes that improve *C. elegans* DMD health of which four are currently approved for use in DMD patients^10^. *C. elegans* is a model for DMD research with numerous clinically relevant phenotypes, increasing the translatability between organisms^10^. The clinically relevant phenotypes include altered gait, decreased maximal voluntary activity, altered neuromuscular signalling, altered excitation-contraction coupling, decreased mitochondrial oxidative capacity, decreased lifespan, muscle cell death, and decreased maximal voluntary contraction strength^10^. In addition, *C. elegans* muscle is both functionally and structurally similar to humans and displays ageing/disease-related deterioration that align with functional read outs^11,12^. Their short lifespan, ease of maintenance and simplistic in vivo genetic manipulability makes *C. elegans* an excellent model for understanding conserved mechanisms of muscular dystrophy pathogenesis. Of note, the dystrophin gene is not X linked in *C. elegans* and therefore the inheritance of DMD is partially different between mammals and worms. Similarly, *C. elegans* do not possess muscle satellite cells or an appreciable inflammatory system, and muscles are mono-nuclear and senescent at adulthood. Thus, while *C. elegans* is an excellent model for some aspects of DMD research, there are aspects that cannot be easily modelled. For this study, we employed a nonsense mutation at position 3287 in the DYS-1 dystrophin orthologue (*dys-1(eg33)*) which induces movement and strength declines as well as intramuscular alterations that correspond with the patient phenotype^13,14^.

Previously, we established that hydrogen sulfide (H_2_S) supplementation via two H_2_S compounds (sodium GYY4137 (NaGYY) and AP39), improved healthspan in *C. elegans dys-1(eg33)* mutants^13^. H_2_S is a conserved mitochondrial substrate and post-translational regulator across species^15^, augmenting anti-inflammatory/oxidative mechanisms at low-moderate concentrations. H_2_S is produced endogenously by three constitutively active enzymes cystathionine-γ-lyase (CSE), cystathionine-β-synthase (CBS) and 3-mercaptopyruavate transferase (3-MST). All three of these enzymes partake in pathways associated with sulfur metabolism including the trans-methylation (TMP) and trans-sulfuration pathways (TSP). L-methionine is converted to L-homocysteine via the TMP using the enzymes s-adenosylmethionine synthetase (SAMS), methyl transferase (PRMT) and s-adenosylhomocysteine hydrolase (AHCY). L-homocysteine can be recycled back to L-methionine via the remethylation pathway (RMP) using the enzyme methionine synthase (METR). CBS and CSE can then catalyse L-homocysteine to homoserine and homolanthionine producing H_2_S as a by-product. L-homocysteine may also be converted to L-cystathionine by CBS and then L-cystathionine is converted to L-cysteine by CSE. L-cysteine is further broken down by the TSP; CBS and CSE catalyse the de-sulfhydration of L-cysteine to produce H_2_S and 3-MST generates H_2_S by modifying the action of the cysteine aminotransferase enzyme (CAT). L-cysteine may also be catalysed to L-glutathione via L-glutathione synthase enzymes and to L-taurine via three other enzymes including cysteine dioxygenase (CDO)^16–18^.

We also recently demonstrated a H_2_S deficiency in dKO (utrophin/dystrophin)-deficient mice and a decline in protein expressions of CSE and 3-MST in these animals^13^. Therefore, we wanted to determine if the *C. elegans dys-1(eg33)* mutant displayed altered sulfur metabolism and if this corresponded with a decline in H_2_S levels. Here, we demonstrate a defect in sulfur metabolism that aligns with reduced global H_2_S levels, both factors significantly restored with NaGYY and AP39 supplementation. Knowing that DMD worms have an NAD defect that can be corrected by NAD supplementation^19^, we tested the beneficial effects of sulfur supplementation via nutrients approved for use in people. We supplemented the sulfur containing amino acids (SAA) that form the basis of this pathway: L-methionine, L-homocysteine, L-cysteine, L-glutathione, and L-taurine. All SAA improved health in *dys-1(eg33)* animals through similar but distinct mechanisms to the aforementioned H_2_S compounds. This study highlights that the method used to supply sulfur is just as important as the sulfur itself, and provides evidence for SAA supplementation as a potential therapeutic in DMD patients.

## Results

### DMD *C. elegans* have altered levels of enzymes associated with sulfur metabolism and have reduced H_2_S levels

**Fig. 1:**
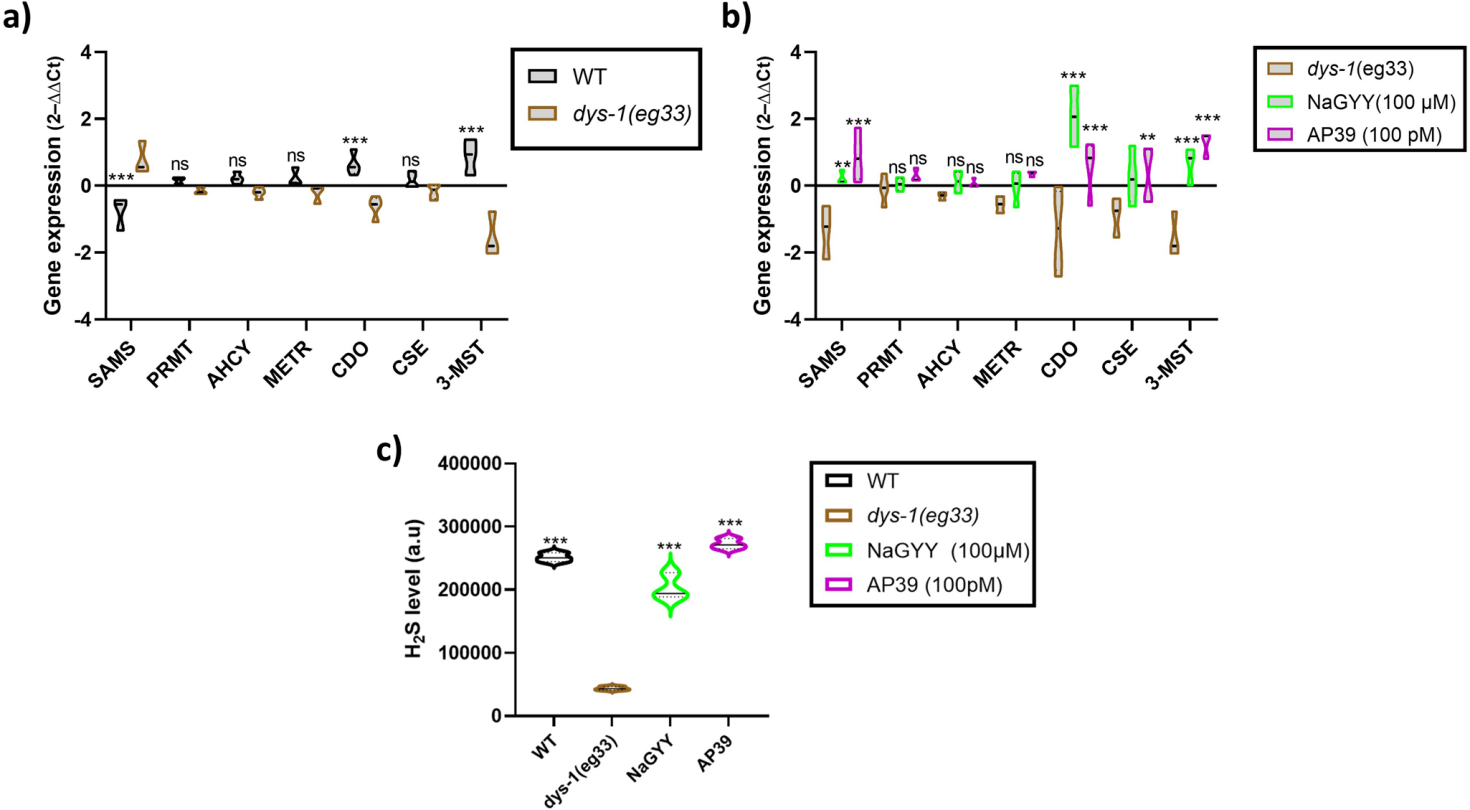
DMD animals have alterations in sulfur metabolism and reduced H_2_S levels. **a** Relative quantification mRNA levels of enzymes associated with sulfur metabolism in *wt* and *dys-1(eg33)* animals. An increase can be seen in SAMS (s-adenosylmethionine synthetase), CDO (cysteine dioxygenase) and 3-MST (3-mercaptopyruavate transferase) in *dys-1(eg33)* animals. **b** Increased expression can be seen in SAMS, CDO, CSE (cystathionine-γ-lyase) and 3-MST with NaGYY and AP39 treatment. There were no differences in PRMT (methyl transferase), AHCY (s-adenosylhomocysteine hydrolase) or METR (methionine synthase). Data are mean ± SD from three biological repeats using 150–200 animals. Results were analysed with a two-way ANOVA. All significance points are compared to *dys-1(eg33)*. ***P < 0.0001, **P < 0.01, *P < 0.05, ns *P* > 0.05. **c** Fluorescence intensity of the AzMC signal, normalised to protein content. There is a decline in global H_2_S levels in the *dys-1(eg33)* animals compared to *wt*, which is increased by NaGYY and AP39 treatment. Data for each treatment are from 270 animals across three biological repeats. Results were analysed with a one-way ANOVA. All significance points are compared to *dys-1(eg33)*. ***P < 0.0001.

Previously, we established for the first time that there was a reduction in sulfur levels and H_2_S producing enzymes in dKO DMD mice^13^. We also demonstrated that supplementation of the H_2_S compounds NaGYY and AP39 could improve health in *dys-1(eg33) C. elegans*, suggesting a sulfur deficit in these animals too^13^. To determine if there were sulfur metabolism alterations in *dys-1(eg33)* worms, we performed RT-qPCR experiments on several enzymes that form part of the sulfur metabolism pathway. We found that *dys-1(eg33)* animals have increased expression of SAMS (responsible for one of the stages to convert methionine to homocysteine) and reduced expression of CDO (responsible for one of the stages to convert cysteine to taurine) and 3-MST (one of the H_2_S generating enzymes) compared to wild-type (*wt*) (Fig. 1a). NaGYY and AP39 treatment of *dys-1(eg33)* animals caused an increased expression of SAMS, CDO, CSE (another H_2_S generating enzyme) and 3-MST (Fig. 1b). No significant differences were observed in PRMT, AHCY, and METR, all of which are part of the TMP and RMP (Fig. 1a, b). These data highlight alterations in sulfur metabolism in *dys-1(eg33)* animals as suspected and that supplementation of H_2_S compounds increases the expression of some of these enzymes. We next wanted to see whether the alterations in sulfur metabolism corresponded with a decline in H_2_S levels. Using a method that employs the H_2_S fluorogenic probe 7-Azido-4-methylcoumarin (AzMC) to measure H_2_S levels in *C. elegans*^20^, we found a decline in global H_2_S levels in *dys-1(eg33)* animals; supplementation with NaGYY and AP39 increased H_2_S levels in these worms (Fig. 1c). We suspect that the increased H_2_S levels from these donors results primarily via upregulation of both CSE and 3-MST expression (Fig. 1b).

### Supplementation of SAA increases H_2_S levels and improves functional defects in movement of *dys-1(eg33)* animals

**Fig. 2:**
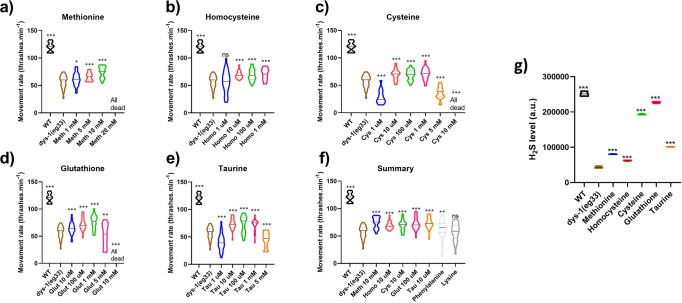
SAA improve movement and increase H_2_S levels in dys-1(eg33) animals. **a** L-methionine treatment significantly improved thrashing rates of *dys-1(eg33)* animals in a dose-dependent manner (1–10 mM). **b** L-homocysteine improved movement in *dys-1(eg33)* (10 µM–1 mM). **c** L-cysteine significantly improvement thrashing in *dys-1(eg33)* (10 µM–5 mM). **d** *dys-1(eg33)* movement was improved with L-glutathione treatment (10 µM–5 mM). **e** L-taurine treatment also improved thrash rate in *dys-1(eg33)* (10 µM–5 mM). **f** Summary figure of the selected optimum doses for each SAA, there are no significant differences between amino acids. Phenylalanine improves movement in the *dys-1(eg33)* model but not to the same levels as the SAA. There is no improvement with lysine treatment. For all strains and treatment, 10 animals were assayed and averages taken from 5 separate counts and repeated across three biological repeats for a total of 150 data points per violin plot. Results were analysed with a two-way ANOVA. All significance points were compared to *dys-1(eg33)* ***P < 0.0001, **P < 0.005, *P < 0.05, ns *P* > 0.05. **g** Fluorescence intensity of the AzMC signal, normalised to protein content. All SAA increased H_2_S levels. Data for each treatment are from 270 animals across three biological repeats. Results were analysed with a one-way ANOVA. All significance points are compared to *dys-1(eg33)*. ***P < 0.0001.

Having established sulfur metabolism defects in *dys-1(eg33)* animals, we were interested to see if supplementing SAA (that are a part of or come off from these pathways) could improve muscle health. We assessed L-methionine, L-homocysteine, L-cysteine, L-glutathione, and L-taurine supplementation in *dys-1(eg33)* thrashing ability (an established indirect measure of muscle health and animal healthspan), to determine effective health-preserving concentrations for each SAA (Fig. 2a–f). We found all amino acids were able to improve movement in a dose-dependent fashion. The following concentrations were used throughout the rest of this study unless otherwise stated: L-methionine 10 mM (Fig. 2a), L-homocysteine 10 µM (Fig. 2b), L-cysteine 10 µM (Fig. 2c), L-glutathione 100 µM (Fig. 2d) and L-taurine 10 µM (Fig. 2e). It is interesting to note that L-methionine was required at a concentration at least 10-fold higher than the other SAA, likely due to methionine’s roles in protein synthesis and direct incorporation into most proteins^21,22^; in other words, there is an increased requirement for methionine for cellular processes beyond the smaller demand for H_2_S metabolism. In addition, there are no major differences to the degree of movement preservation between various SAA (Fig. 2f), indicating all SAA are effective health-preserving compounds in *dys-1(eg33)* mutants. We also trialled two non-sulfur containing amino acids: phenylalanine and lysine. Phenylalanine gave a slight improvement in movement (*P* < 0.005) but not to the same levels as the SAA, where lysine showed no improvements (Fig. 2f). These data suggest the sulfur component underlies the health-preserving effects, corresponding with our previous data from NaGYY and AP39^13,14^. Having seen this improvement, we also assessed whether SAA increased global H_2_S levels. All SAA increased H_2_S in *dys-1(eg33)* animals but to different degrees. Unsurprisingly, L-cysteine gave one of the largest improvements as this is the main substrate for the H_2_S-generating enzymes (Fig. 2g).

### SAA supplementation delays the onset of muscle cell death and improves mitochondrial structure

**Fig. 3:**
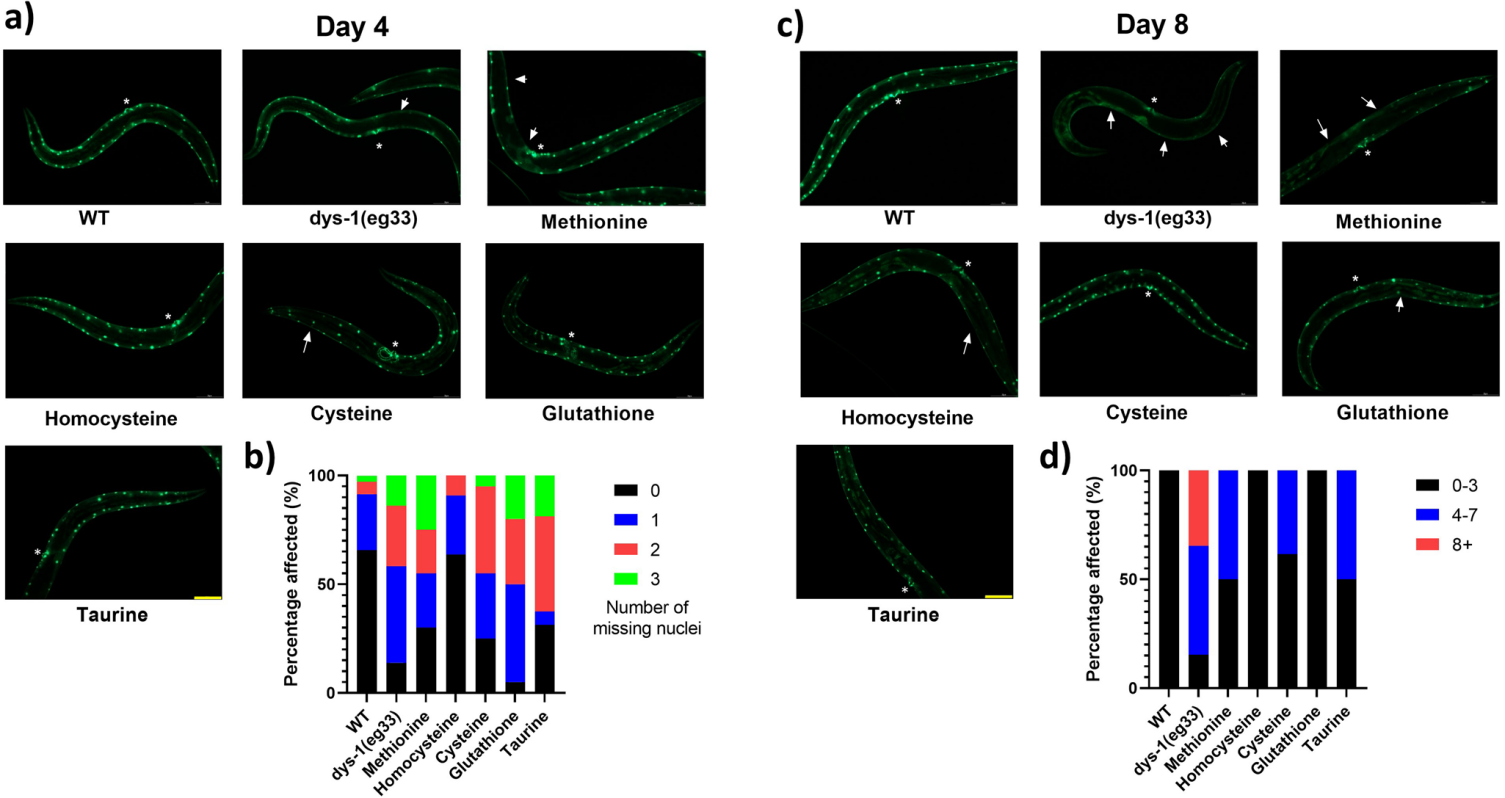
SAA mildly delay onset of cell death at Day 4 post-adulthood, with significant preservation at Day 8. **a** Representative images of muscle cell death in CB5600 (*wt*), CC91 (*dys-1(eg33)*) and CC91 treated with SAA: L-Methionine (10 mM), L-homocysteine (10 µM), L-cysteine (10 µM), L-glutathione (100 µM) and L-taurine (10 µM). Muscle cell death (as identified by the absence of muscle nuclei) was more pronounced in the *dys-1(eg33)* animals compared to *wt*. **b** The scatter bar graph shows a higher proportion of animals with no missing nuclei when treated with SAA bar L-glutathione. However, the proportion of animals with 2 or more missing nuclei was similar across all groups bar L-homocysteine. **c** Representative images of muscle cell death in CB5600 (*wt*), CC91 (*dys-1(eg33)*) and CC91 treated with SAA: L-Methionine (10 mM), L-homocysteine (10 µM), L-cysteine (10 µM), L-glutathione (100 µM) and L-taurine (10 µM). Muscle cell death was more pronounced in the *dys-1(eg33)* animals compared to *wt* at Day 8 post-adulthood. **d** The scatter bar graph shows a higher proportion of animals with 0–3 missing nuclei when treated SAA in particular, L-homocysteine and L-glutathione. Vulva is identified by the * and arrows show the missing nuclei. Scale bar: 30 µm. Data are means from 20 animals across two biological repeats.

Given the functional improvements in muscle health demonstrated above, we wondered how SAA impacted the sub-cellular pathology of DMD in *C. elegans*. Quantitative time course electron microscopy demonstrates that there is a histological progression of DMD in *C. elegans*^23^. First, muscle attachment complexes fail, followed by loss of sarcolemma integrity, sarcoplasmic reticulum structure, mitochondrial structure, sarcomere integrity and ultimately loss of nuclei and muscle cell death^23^. Taking advantage of the fact that loss of well organised muscle nuclei can be observed via GFP tagged nuclei to identify muscle cell death in DMD worms^24^, we began by examining the impact of SAA supplementation on muscle cell death. At Day 4 of adulthood, *dys-1(eg33)* mutants start to display muscle cell death with most worms showing at least one missing muscle nuclei. The SAA treated animals show a slightly higher percentage of animals with no missing nuclei compared to untreated *dys-1(eg33)*, however the proportion of animals with two or more missing nuclei was similar across all groups bar L-homocysteine (Fig. 3a, b). As cell death is a progressive feature of DMD, we wondered if suppression of cell death was more evident with later age, as we have previously shown with NaGYY^13^. In agreement with the published NaGYY data, we find that all SAA offset cell death seen in *dys-1(eg33)* mutants at Day 8, with best preservation observed with L-homocysteine and L-glutathione supplementation (Fig. 3c, d).

**Fig. 4:**
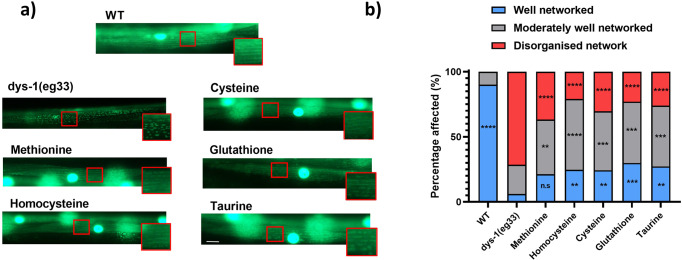
The mitochondrial network of dys-1(eg33) animals is improved by SAA treatment. **a** Representative images of CB5600 (*wt*) with GFP tagged mitochondria which displayed a well organised mitochondrial network. CC91 (*dys-1(eg33)* with GFP tagged mitochondria which showed a severely disorganised mitochondrial network. L-homocysteine (10 µM), L-cysteine (10 µM), L-glutathione (100 µM) and L-taurine (10 µM), all significantly improved the proportion of well-networked mitochondria, and also reduced the degree of disorganised networks to *dys-1(eg33)* animals, but not to *wt* levels. L-Methionine (10 mM) did not significantly increase well-networked proportions, but did offset the prevalence of disorganised networks to *dys-1(eg33)*. **b** A stacked bar graph showing the percentage of well-networked, moderately well-networked and disorganised network within each group. Scale bar: 30 µm. Data are means from 20 animals across two biological repeats.

As loss of mitochondrial integrity appears to result in cytochrome C release to activate cell death in *C. elegans* DMD muscle^25^, we next assessed mitochondrial structure in response to SAA supplementation and compared this with results from H_2_S supplementation via NaGYY and AP39^13^. We classified the appearance of mitochondria from each worm into one of three groups: well networked, moderately well-networked and disorganised network as described previously^13^ and were able to confirm significant improvements in mitochondrial structure with SAA supplementation (Fig. 4a, b). *dys-1(eg33)* animals displayed significant declines in well-networked mitochondria (−84.04%, *P* < 0.0001) and an increased prevalence of severely fragmented mitochondria (+71.41%, *P* < 0.0001) to *wt*. Importantly, supplementation of all SAA (except methionine) significantly increased the proportion of well-networked mitochondria in *dys-1(eg33)* (L-methionine = +15.20% (*P* > 0.05), L-homocysteine = +18.60% (*P* < 0.01), L-cysteine = +18.21% (*P* < 0.01), L-glutathione = +23.78% (*P* < 0.001) and L-taurine = +21.14% (*P* < 0.01)). Despite an insufficient significant increase in well-networked mitochondria with L-methionine, there was a significant decline in the presence of disorganised networks compared to *dys-1(eg33)* (−34.66%, *P* < 0.0001). This effect was also clear with all other SAA supplementation (L-homocysteine = −50.47% (*P* < 0.0001), L-cysteine = −41.03% (*P* < 0.0001), L-glutathione = −48.33% (*P* < 0.0001) and L-taurine = −45.32% (*P* < 0.0001)). Therefore, these data suggest that modulation of SAA metabolism can reduce impaired mitochondrial networking in *dys-1(eg33)* in vivo. While the increased proportions of well-networked mitochondria are mild, the shifts from disorganised mitochondria towards moderate fragmentation imply an important preservation of mitochondrial integrity.

### Mitochondrial dysfunction is better reversed by mitochondrial targeted H_2_S supplementation than by SAA supplementation

**Fig. 5:**
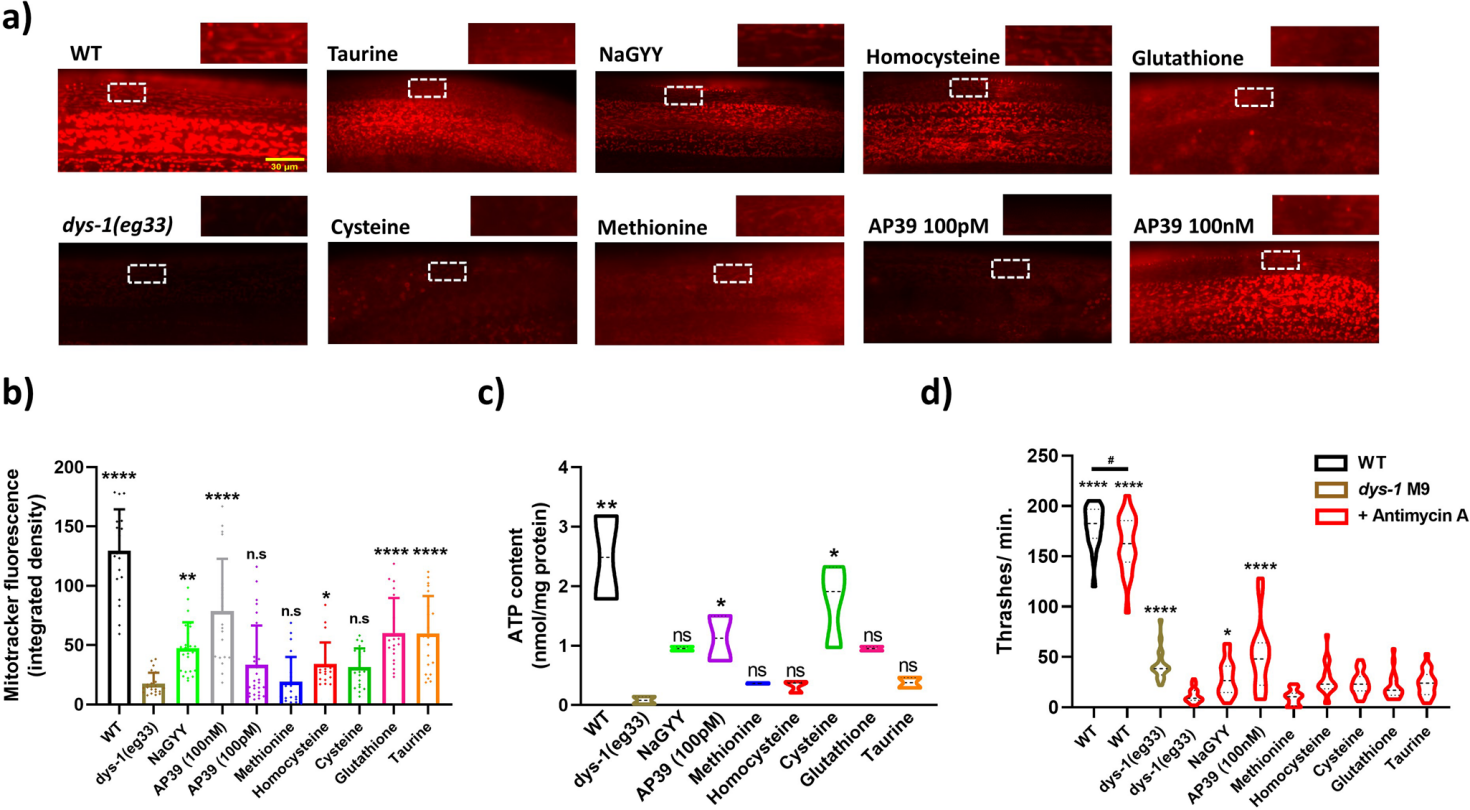
H_2_S compounds show greater mitochondrial function improvements to SAA in dys-1(eg33) mutants. **a** Representative images of mitotracker fluorescence uptake. Cut away images are of mitochondria within body-wall muscle to highlight fluorescence signals. Scale bar: 30 µm. **b** Quantification of Mitotracker fluorescence. Untreated *dys-1(eg33)* animals exhibit significant declines in mitochondrial membrane potential to *wt*. Treatment with 100 μM NaGYY and 100 nM (but not 100 pM) AP39 were able to significantly improve membrane potential. In addition, L-taurine, L-homocysteine and L-glutathione (but not L-cysteine and L-methionine) were able to significantly augment mitochondrial membrane integrity in *dys-1(eg33)* mutants. Data are mean + SD of two biological repeats, with 50–60 animals per condition. Asterisks denote significance to untreated *dys-1(eg33)* animals (*P < 0.05, **P < 0.01, ****P < 0.0001). **c** There is a decline in ATP content in *dys-1(eg33)* animals. Treatment with AP39 (100 pM) and L-cysteine significantly increased ATP content. The other SAA trialled did not induce a significant improvement in ATP content. Data for each treatment are from 270 animals across three biological repeats. **P < 0.01, *P < 0.05. **d** Complex III inhibition induces significant movement decline in *dys-1(eg33)* that is restored with AP39 (100 nM) and NaGYY administration, but not SAA. Data are obtained from two biological repeats with 30–40 animals per condition. Asterisks denote significance to *dys-1(eg33)* + Antimycin A exposure (*P < 0.05, ****P < 0.0001). ‘#’ denote significance between *wt* treatments (^#^*P* < 0.05).

Given the similar healthspan preserving effects of exogenous H_2_S and SAA supplementation, we were curious if exogenous H_2_S and SAA supplementation had similar improvements in mitochondrial function. DMD *C. elegans* display decreased oxidative capacity that is improved with H_2_S supplementation^13,14^. This decline in function is accompanied by impaired mitochondrial membrane potential^13,14^ and by increased sensitivity to electron transport chain inhibitors^13^. Therefore, we examined the ability of the various treatments to improve mitochondrial membrane potential. We exploited the cationic dye MitotrackerCMXRos, with a mildly thiol-reactive chloromethyl moiety for mitochondrial labelling to assess membrane potential in situ. Consistent with past reports^13,14^, we observe that *dys-1(eg33)* mutants display impaired mitochondrial membrane potential compared to *wt*, as noted by reduced fluorescence intensity and localisation of the dye within body-wall mitochondria (Fig. 5a, b). Both forms of H_2_S supplementation improved membrane potential whereas only some of the SAA supplements significantly improved membrane potential (Fig. 5a, b); the health-preserving concentrations of L-taurine, L-homocysteine and L-glutathione improved membrane potential. These results suggest that the SAA supplementation is not working exactly the same as H_2_S supplementation. However, as our doses for supplementation are based upon movement increase it is possible that higher levels of supplementation might improve mitochondrial function despite not further improving overall movement capacity. For example, the 100 pM dose of AP39 which is sufficient to improve whole animal movement, does not display a significant increase in mitochondrial membrane potential, whereas 100 nM does (Fig. 5a, b); note that 100 nM also improves movement to the same extent as 100 pM^13^.

We next measured ATP content of the *dys-1(eg33)* animals with the various treatments (Fig. 5c). As anticipated, there is less ATP content in the *dys-1(eg33)* animals compared to *wt*, confirming a consequence of mitochondrial dysfunction in these animals. Only AP39 and L-cysteine supplementation displayed a significant increase in ATP content. The result for AP39 is unsurprising as it is targeted directly to the mitochondria, but the L-cysteine-mediated increase is interesting as it is not targeted. However, L-cysteine is the main SAA precursor of H_2_S. This demonstrates that the delivery method for supplementing sulfur is important for the effects it can have on muscle health.

Lastly, we examined the ability of the various treatments to reverse the documented Antimycin A hypersensitivity in *dys-1(eg33)* worms^13^. As shown in Fig. 5d, we were able to observe increased sensitivity to the electron transport chain complex III inhibitor and this was reversed by supplementation with exogenous H_2_S, as previously reported^13^. In contrast, we found no significant reversal of hypersensitivity to electron transport chain inhibition with SAA exposed animals, despite a trend for improvement (Fig. 5c). Thus, SAA supplementation does not appear to improve mitochondrial function to the same extent as H_2_S supplementation.

### SAA and mitochondrial H_2_S differentially affect mitochondrial superoxide production

**Fig. 6:**
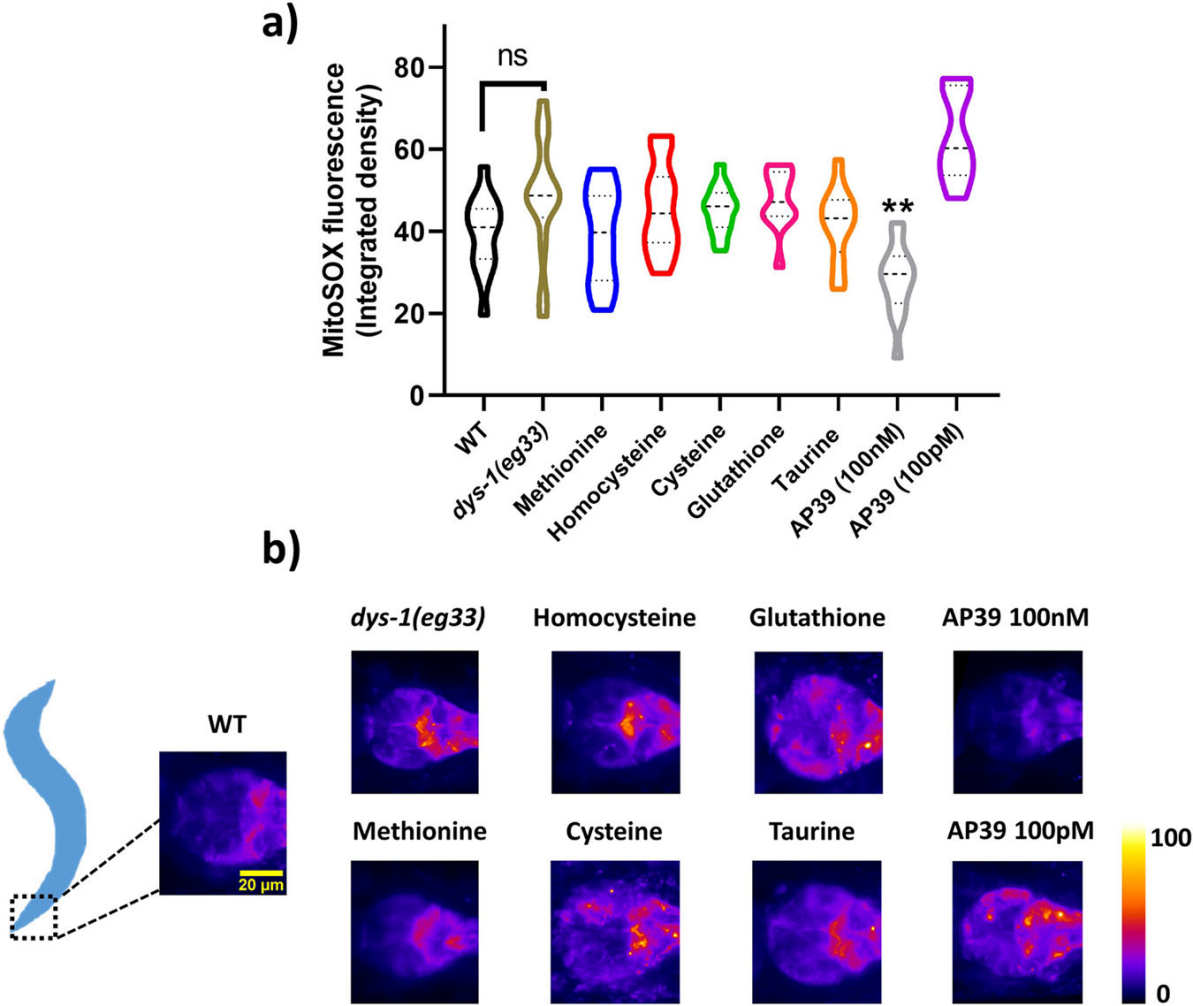
Mitochondrial superoxide production in dys-1(eg33) mutants is not altered with SAA exposure. **a** *dys-1(eg33)* animals do not display a significant increase in MitoSOX fluorescence within the terminal bulb of the pharyngeal muscles compared to *wt* animals, despite a visual trend for increased fluorescence intensity. SAA did not alter the fluorescence signal, however, 100 nM AP39 significantly lowered mitochondrial superoxide (but not 100 pM) to *dys-1(eg33)* controls. Scale bar: 20 µm. **b** Representative images using a fire LUT to enhance visual clarity of MitoSOX fluorescence. Data are presented as violin plots to display the distribution of the data. Dashed lines represent the median and quartiles are represented by the dotted lines. Data are obtained from two biological repeats with 20–30 animals per condition. Asterisks denote significance to *dys-1(eg33)* control animals. **P < 0.01, ****P < 0.0001.

It is well known that mitochondrial fragmentation and muscle dysfunction are associated with elevated levels of reactive oxygen species (ROS)^26^. While mitochondrial ROS are fundamental to healthy ageing, supra-physiological concentrations of ROS are well known to cause deleterious effects, such as irreversible over-oxidation and loss of protein function^15^. Since H_2_S and SAA supplementation both improve mitochondrial structure in DMD worms, we were curious if differences in the restoration of mitochondrial function were due to alterations in mitochondrial ROS levels. While there was a trend for increased ROS, we observed no significant increases in superoxide production in the pharyngeal muscles in the *dys-1(eg33)* animals (Fig. 6a, b). Therefore, it is unsurprising that supplementation of SAAs did not significantly decrease superoxide levels. In addition, we saw only slightly detectable superoxide levels in NaGYY exposed DMD animals. Upon closer observations, we noted that the combination of NaGYY with MitoSOX on NGM plates results in direct quenching of the fluorogenic properties of this probe, meaning the animals were not exposed to sufficient amounts of MitoSOX in this co-treatment method (Supplementary Fig. 1). DMD animals supplemented with mitochondrial targeted H_2_S displayed no quenching of the MitoSOX probe and a significant decline in ROS. Just as AP39 displayed a dose-dependent effect on mitochondrial membrane potential, it also displayed a dose-dependent effect on mitochondrial ROS levels. Thus, H_2_S and SAA appear to differentially affect mitochondrial ROS production, much as they differentially affect mitochondrial function. It must be noted, that the MitoSOX fluorogenic probe utilised here exploits a positively charged triphenylphosphonium linker (to sequester preferentially within mitochondria given their electrochemical oppositions). Therefore, as DMD worms have impaired mitochondrial membrane potential, the lack of observed ROS elevation may be explained by an inability of the probe to accumulate within mitochondria themselves.

### SAA but not H_2_S supplementation improves muscle calcium handling and excitation-contraction coupling in DMD worms

**Fig. 7:**
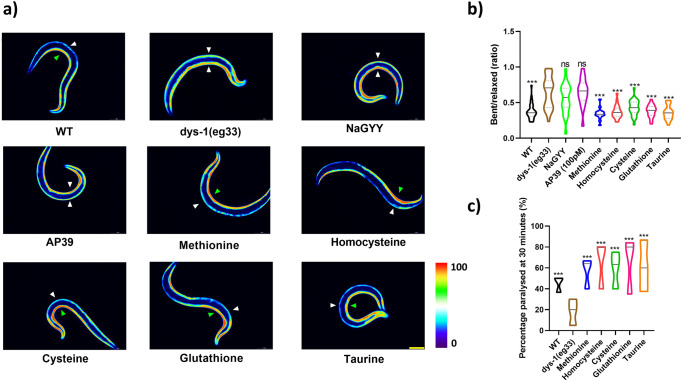
Calcium mishandling is improved in the dys-1(eg33) animals treated with SAA. **a** Representative images of calcium report strain in HBR4 (*wt*), HBR4xBZ33 (*dys-1(eg33*)) and HBR4xBZ33 treated with H_2_S and SAA: L-methionine (10 mM), L-homocysteine (10 µM), L-cysteine (10 µM), L-glutathione (100 µM) and L-taurine (10 µM). In *wt* animals, high levels of calcium can be detected on the contracted side (green arrow) of the body-wall muscles with no calcium being detected on the relaxed side (white arrow). In *dys-1(eg33)* animals, high levels of calcium can be detected on both the relaxed and contracted sides. Treatment with SAA restored calcium handling to that which represented *wt*. **b** Quantification of the relative ratio of calcium intensity for bent (contracted) side vs relaxed side. The ratio is higher than *wt* in *dys-1(eg33)* and is restored by treatment with SAA. Both NaGYY and AP39 were unable to confer significant improvements in calcium handling. Data are from 20 animals across two biological repeats. Scale bar: 30 µm. **c** *dys-1(eg33)* animals are resistant to levamisole after 30 min of exposure compared to *wt*. This is improved by all SAA. Data are from 30 animals across three independent biological repeats. Results were analysed with a two-way ANOVA. All significance points were compared to *dys-1(eg33)*. ***P < 0.0001, ns *P* > 0.05.

One of the main underlying pathophysiological mechanisms in DMD is mishandling of calcium and calcium overload^3,27^. We therefore wanted to determine whether SAA could improve calcium handling in vivo. First, we crossed HBR4 (a reporter strain which expresses the calcium indicator GCaMP3 in all body-wall muscles) with the *dys-1(eg33)* strain. Muscle contraction requires a sufficient calcium current, where a rise in calcium can be seen on the contracted (but not relaxed) side in *wt* animals (Fig. 7a, green arrow contracted side, white arrow relaxed side). Conversely, in *dys-1(eg33)* mutants, elevated calcium can be seen on both the contracted and relaxed sides regardless of muscle contraction state (Fig. 7a, both white arrows). When we supplemented *dys-1(eg33)* animals with any of the SAA we saw a restoration of more normal calcium handling, in that there were high levels of calcium on the contracted side of body-wall muscles and the elevated levels of calcium on the relaxed side were no longer as apparent (Fig. 7a). Quantification of contraction vs relaxation ratios confirmed that SAA supplementation improved calcium handing (Fig. 7b).

Having observed an improvement in calcium handling with the calcium reporter strains we next examined levamisole sensitivity. Previously, DMD worms have been shown to be resistant to levamisole, indicative of defective post-synaptic excitation-contraction coupling^14^. We supplemented *dys-1(eg33)* animals with SAAs and assessed their resistance to levamisole (Fig. 7c). We observed an increase in sensitivity to levamisole with all SAAs, implying that SAA supplementation can improve the defects in post-synaptic excitation-contraction coupling (Fig. 7c). Taken together these data suggest that SAA can improve calcium dysregulation in DMD. This contrasts with H_2_S supplementation, where no improvement in excitation-contraction coupling is observed^13^. Combined, these results suggest that there are multiple consequences to sulfur depletion in DMD muscle and reinforce the observation that the method of sulfur supplementation influences the impact(s) on muscle health.

### Genes required for SAA-mediated healthspan preservation are similar to those required for H_2_S supplementation

**Fig. 8:**
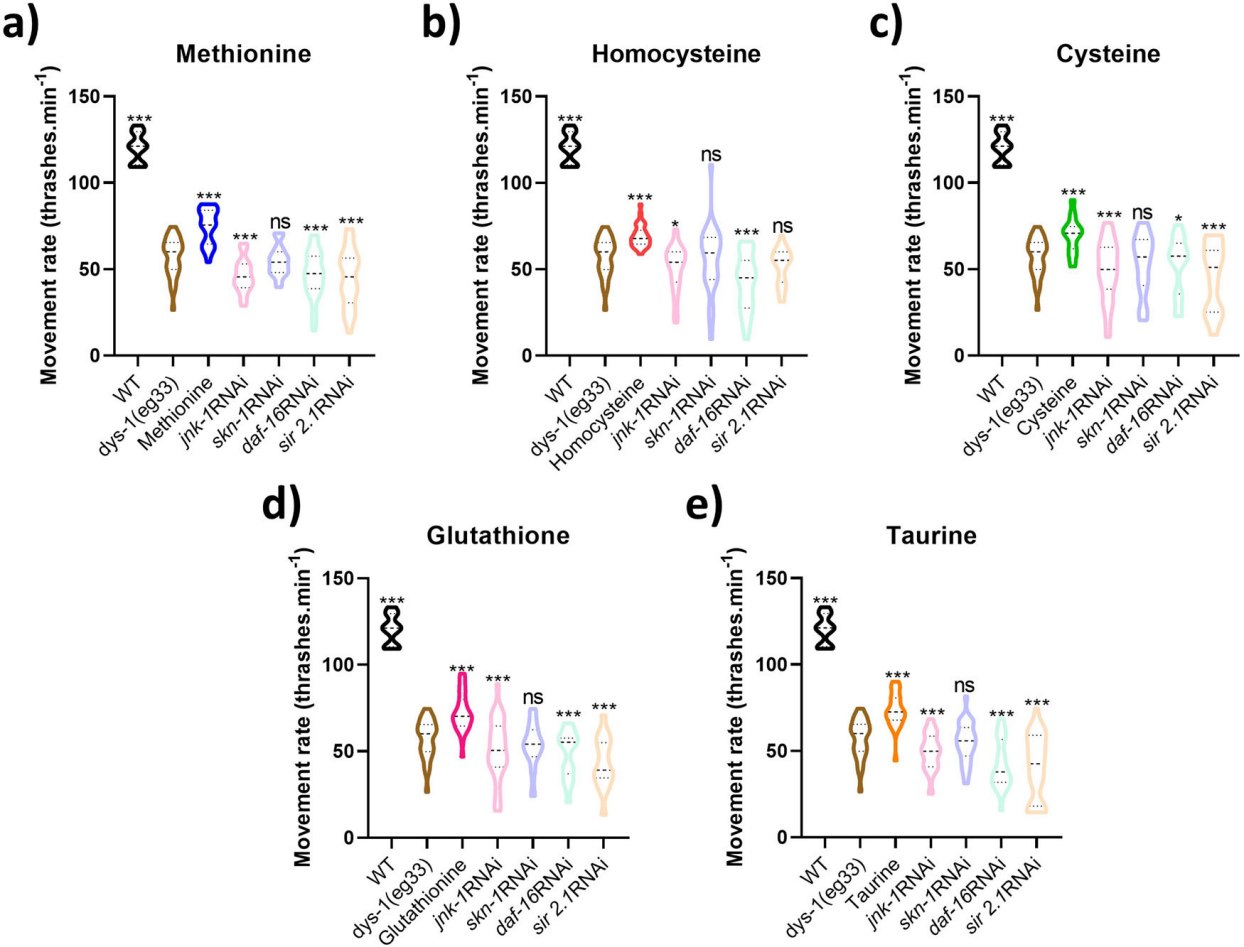
Conserved cytoprotective genes are required for the health promoting effects of SAA. **a** L-methionine, **b** L-homocysteine, **c** L-cysteine, **d** L-glutathione and **e** L-taurine all require *jnk-1, skn-1, daf-16* and *sir-2.1* for their established health promoting effects on *dys-1(eg33)* movement capacity. Despite *skn-1* co-treatments not presenting a significant decline in movement compared to untreated *dys-1(eg33)*, the lack of movement preservation (as seen with the compounds alone) suggests each SAA requires full expression of this gene. All strains and conditions represent data from 10 animals with averages taken from 5 separate counts and repeated across three biological repeats. Results were analysed with a two-way ANOVA. Asterisks denote significance to untreated *dys-1(eg33)* animals. ***P < 0.0001, *P < 0.05, ns *P* > 0.05.

In our previous study we identified genes that appear to be required for the beneficial health effects of H_2_S compound supplementation in DMD worms^13^. Like H_2_S compound supplementation in DMD worms, we have seen an improvement in both cell death and mitochondrial structure with SAA supplementation. Thus, we were interested to see if the genes with known requirement for NaGYY’s healthspan effects in DMD were also required for the beneficial effects of SAA supplementation^13^. Supplementation with all SAA required the stress responsive protein kinase JNK-1 and the stress and metabolic transcription factor DAF-16 for their beneficial effects (Fig. 8a–e). Supplementation with all SAA appear to require the deacetylase SIR-2.1 present for beneficial locomotory effects. Despite the SIR-2.1 induced movement decline with L-homocysteine not reaching significance, the repression of L-homocysteine’s full healthspan effects suggests some involvement for SIR-2.1 in regulating this compounds response (Fig. 8b). Interestingly, the transcription factor SKN-1 displays similar conditions to SIR-2.1’s regulation of L-homocysteine’s effects, in that its expression appears partly required for all SAA, but to a lesser degree than the other transcriptional regulators within our screen (Fig. 8a–e). This is particularly striking as *skn-1* is an ortholog of NrF2, a known regulator of oxidative stress and a known target for the effects of H_2_S in mice^28^.

## Discussion

DMD is a severe, genetically inherited disorder culminating in muscle degeneration, respiratory impairment, and ultimately death. There is an urgent unmet clinical need for drug-based countermeasures. Previously, we showed that H_2_S administration in *dys-1(eg33)* mutants restored mitochondrial function, animal strength and overall muscle health^13^, implying a potential deficit in H_2_S producing pathways in these mutants (as demonstrated in *mdx* mice^13^). Here, we confirm a dysregulation in expression patterns of genes involved in sulfur metabolism which decreases H_2_S levels in *dys-1(eg33)* mutants. SAA administration increased H_2_S levels and improved animal health. These improvements appear to act largely via improved calcium handling and excitation-contraction coupling when compared to H_2_S donor compounds. As calcium dysregulation is an established phenomenon that can cause mitochondrial dysfunction^29^, SAA supplementation may act, in part, by reducing a ‘calcium-first’ mechanism of cellular degeneration in DMD, augmenting mitochondrial health as a secondary consequence.

### Cause and consequences of sulfur deficit in DMD muscle

The results of the present study confirm that like rodent DMD muscle^13^, worm DMD muscle display a sulfur deficit. The alterations in sulfur metabolism pathway genes suggest that DMD muscle responds to this deficit by adjusting sulfur metabolism. Given that mitochondrial sulfur supplementation is sufficient to improve mitochondrial and animal health^13^, it is likely that the sulfur metabolism deficit in DMD muscle is caused by an increased demand for sulfur in the mitochondria. Two well-established targets of H_2_S are in the mitochondria, cytochrome *c* oxidase^30^ and ATP synthase^31^. Thus, it could be that the increased demand of sulfur in the mitochondria is to maintain electron transport chain function. Regardless of the precise cause, the consequence of this increased need for sulfur within mitochondria is a decline in sulfur availability for other purposes. For example, our results imply SAA deficits in DMD muscle. These have previously been reported in rodent DMD muscle for cystine and taurine^32^. It could be that some elements of loss of homeostasis in DMD muscle are underpinned by SAA deficits. For example, DMD muscles display decreased protein synthesis^33^, is this partially underpinned by a methionine deficit? Similarly, does the taurine deficit exacerbate the calcium perturbations? Our results with taurine supplementation are particularly suggestive in this regard as taurine supplementation significantly improves calcium handling in DMD muscle. Clearly, further understanding the consequences of the sulfur deficit(s) in DMD muscle is an area for investigation. In contrast, the importance of further understanding the cause of the sulfur deficit in DMD seems less clear. It could simply be a consequence of an inability to maintain mitochondrial membrane potential due to elevated sarcoplasmic calcium levels resulting from a lack of dystrophin and mislocalised calcium channels^34^.

### Clinical implications of the sulfur deficit in DMD muscle

Having established a sulfur deficit is present in worm and rodent DMD muscle, the obvious clinical questions are: (i) how can we use sulfur to treat DMD; and (ii) are sulfur levels a good prognostic marker? Our previous study established that exogenous H_2_S can improve clinical symptoms of DMD in worms^13^. Extension of this finding into rodents and patient derived models are needed. However, the path to clinical approval of new compounds is lengthy. The present study establishes that natural compounds, SAA, can also be used to improve clinical symptoms of DMD worms. Taurine supplementation has also been shown to be effective in DMD rodents^35^. Thus, direct SAA supplementation studies in DMD patients seems viable and warranted. Similar arguments have been made for other diseases such as neurodegeneration^36^, cardiovascular disease^37^, and cancer^38^. Also, in our previous study^13^ we established a correlation between extent of sulfur metabolism disruption and clinical severity in rodents. Those observations coupled with the present observations that low sulfur levels can be treated by supplementation and these correspond with health improvement suggest that direct examination of sulfur levels and severity of DMD and/or progression of DMD is warranted. Should sulfur levels be a good prognostic marker they could be used for clinical decision making beyond just when and how much sulfur to supplement. For example, they could be used to monitor efficacy of other treatments such as corticosteroids.

## Conclusion

Our data show that administration of SAA preserves healthspan in a *C. elegans* model of DMD. When comparing the health-preserving effects of each SAA against gold standard H_2_S compounds AP39 and NaGYY, we show a propensity for improved calcium handling and excitation-contraction coupling mechanisms with SAA vs mitochondrial-predominant patterns for H_2_S compounds as previously shown^13^. Despite this, similar genes are required for both SAA and H_2_S donor healthspan effects, highlighting the multi-purpose role of these pathways. Importantly, SAA treatment presents a safe and immediately exploitable therapeutic strategy for DMD within the clinic that should be explored further in higher DMD models.

## Methods

### Strains and culture conditions

Strains obtained from the *Caenorhabditis* Genetics Center (CGC) were N2 (*wt*) and BZ33 *(dys-1(eg33)*. Mitochondrial network integrity and cell death was assessed using CB5600 [*ccIs4251 (Pmyo-3::Ngfp-lacZ; Pmyo-3::Mtgfp) I; him-8 (e1489)* IV] and CC91 [*dys-1(eg33) I; ccIs4251 I; him-8(e1489)* IV] (developed previously in this lab). The calcium sensor strain used was HBR4 {*goeIs3 [myo-3p::GCamP3.35::unc54 39utr* + *unc-119*(+)]} (kindly donated by Professor Higashitani) and this was crossed in this lab with BZ33 to give the strain HBR4xBZ33. All worms were cultured at 20 °C on NGM agar plates seeded with *Escherichia coli* OP50. For all experiments, animals were age synchronised by washing plates with 2 mL of M9 buffer, where L1 stage animals were collected from the top of 2 mL eppendorphs after 2.5 min of gravity separation allowing older animal displacement towards the bottom of the tube. L1 stage animals were then placed onto 3 cm NGM plates ± compounds and exposed until young adult stages where animals were then collected for experimentation.

### Pharmacological compounds

NaGYY was synthesised as described previously^39^, the compound was dissolved in ddH_2_O and added to the surface of a 3 cm seeded NGM plate approximately 24 h before use. The concentration used was 100 µM. AP39 was synthesised as described previously^40^, the compound was initially dissolved in DMSO and further diluted in ddH_2_O before being dispensed onto the surface of a 3 cm seeded NGM plate approximately 24 h before use. The concentrations used were 100 pM and 100 nM. Both concentrations were determined previously by thrash assay^13^. All SAA were diluted initially in ddH_2_O before being added to the surface of a seeded 3 cm NGM agar plate. Optimal concentrations were determined by thrash assay and are as followed: L-methionine (Sigma-Aldrich, M9625) 10 mM, L-homocysteine (Sigma-Aldrich, 69453) 10 µM, L-cysteine (Sigma-Aldrich, 168149) 10 µM, L-glutathione (Sigma-Aldrich, PHR1359) 100 µM, and L-taurine (Sigma-Aldrich, T0625) 10 µM.

### H_2_S detection in *C. elegans*

H_2_S levels was determined through incubation with the fluorogenic probe 7-Azido-4-methylcoumarin (AzMC) (Sigma-Aldrich, 802409). Briefly, 90 Day 1 adults were picked into M9 buffer, washed three times to remove bacterial debris and left in a final volume of 40 µL. Three freeze-thaw cycles were performed in liquid nitrogen and samples were then centrifuged at 14,000 RPM at 4 °C for 15 min to pellet animal debris. 30 µL of sample supernatant was added to 70 µL of dilute AzMC for a final concentration of 50 µM in 100 µL within each well. Plates were incubated for 2 h at 20 °C in the dark and fluorescence read on a BMG fluorostar plate reader with excitation and emissions of 355/460 nm, respectively. Samples were normalised to protein content determined via the standard Bradford assay^41^.

### mRNA quantification via RT-qPCR

Total RNA was extracted from approximately 150–200 Day 1 nematodes in triplicate using the TRIzol method as previously described^42^. Reverse transcription to cDNA was carried out using oligo(dT) priming according to manufactures protocol (PrimeScript^TM^ RT Reagent Kit, Takara). Real-time PCR was performed using TB Green Premix Ex Taq^TM^ II (Takara) on a CFX Connect^TM^ Real-Time PCR Detection System (Bio-Rad). Relative fold change was determined by 2^-ΔΔCT^ method and normalised to housekeeping gene, *eef-2*. Primer sequences can be found below (Table 1) and were selected from the ORFeome project^43^.

**Table 1:** Primers used in RT-qPCR.

Gene	Forward primer	Reverse primer
*sams-1*	TGTCCTCCAAATTCCTTTTCACC	AGTGAGCGATAGCAGATGT
*prmt-1*	TGAGTACCGAAAACGGGA	AGTGCATGGTGTAGGTGT
*ahcy-1*	TGGCCCAGTCTAAGCCAGCTTAC	AATATCTGTAGTGGTCTGGCTTGT
*metr-1*	TGACTCGAAGTAGTCTTTTCGAGG	AATCCGTATCATAGCCAAGAATTGGT
*cdo-1*	TGATGTTAGTTGTTCAAATTCGTGAAA	AATTGCCATTCTTAGATCCTCTGT
*CSE-2*	TGGCTACTTTCCCACACT	ATACTTTTGGAATGGCAATCTTCA
*3-MST-4*	CCTGTCTTTTGCCTGCCTAC	GCAGAACAATTGAAGCGACA
*eef-2*	TGGTCAACTTCACGGTCGAT	CATCTTGTCGAGATAGTTGTCAAGG

### Thrash assay

The motility of *C. elegans* was assessed by picking 30 individual animals into a liquid medium (M9 buffer) and counting the frequency of thrashes. The number of bends in 10 s was counted and repeated five times for each worm for three independent biological replicates. These were then multiplied by six to give the movement rate per minute. One body bend was recorded as one rightward body bend and leftward body bend. For each treatment, movement rates for ten worms were measured with three biologically independent repeats.

### Mitochondrial and cell death imaging

To assess the mitochondrial network, the CB5600 [*ccIs4251 (Pmyo-3::Ngfp-lacZ; Pmyo-3::Mtgfp) I; him-8(e1489)* IV] strain and CC91 [*dys-1(eg33) I; ccIs4251 I; him-8(e1489)* IV] strain were used for *wt* imaging and dystrophy imaging respectively, that uses myo-3p to express mitochondrial and nuclear GFP within all body-wall muscle cells. Worms were imaged at ×40 magnification using a Nikon Eclipse 50i microscope with images taken from the head and tail regions of each animal. Approximately 20 worms were imaged per strain, having been grown to Day 1 of adulthood either with or without SAA exposure. Animals were then classified into three mitochondrial network categories: well-networked, moderately networked and disorganised networks by visual analysis of mitochondrial morphology within each observable muscle cell, formulating individual percentages for each mitochondrial category, per worm. The same strains were used to evaluate cell death as described previously^13^. Briefly, images were taken on Day 4 and Day 8 of adulthood using a Nikon Eclipse 50i microscope at ×10 magnification. After, manual counting of muscle cells for absent/aggregated GFP signals was performed per animal. *C. elegans* body-wall muscle cells are mononucleate, therefore, loss of observable nuclei is indicative of cell death as previously shown^24^. Approximately 20 animals were assessed per condition.

### Calcium imaging

The calcium concentration in the body-wall muscles of *C. elegans* was determined from HBR4 {*goeIs3 [myo-3p::GCamP3.35::unc54 39utr* + *unc-119*(+)]} and HBR4xBZ33 animals. These animals were generated in our lab by mating HBR4 with BZ33. Approximately 20 animals per condition were imaged on a Nikon Eclipse 50i microscope at ×10 magnification. The intensity of the green fluorescent protein (GFP) was quantified using ImageJ (National Institutes of Health, Bethesda, MD, USA) software.

### Levamisole assay

Approximately 30 Day 1 adult worms were picked into 2.5 mL of levamisole hydrochloride (Sigma-Aldrich, 31742) at 100 µM in M9 buffer. The percentage of animals paralysed at 30 min was recorded. For each experiment there were three biological independent repeats.

### Development of animals on RNAi

Clones were obtained from the Open Biosystems Vidal Library. Clones used were as follows: *jnk-1*: B0478.1, *skn-1*: T19E7.2 and *daf-16*: R13H8.1. *sir-2.1*: R11A8.4 clone was obtained from the Ahringer library. The RNAi feeding method was utilised in this case, where worms were fed bacteria expressing dsRNA^44^. L1 worms were synchronised as described previously onto RNAi plates containing a bacterial lawn expressing one of the RNAis. Worms were left to develop at 20 °C until Day 1 of adulthood where a thrash assay was then carried out.

### ATP content

ATP content was determined using the CellTiter-Glo 2.0 assay (Promega) with ATP standard curve (Sigma) as described previously^45^. A total of 90 animals were picked into M9, washed three times with M9 to remove bacterial debris and then snap-frozen in liquid nitrogen at a final volume of 30 µL. This was followed by three freeze-thaw cycles and samples were then centrifuged at 14,000 RPM at 4 °C for 15 min to pellet animal debris. Supernatants were collected where 8 µL of sample was diluted in 152 µL of M9. 50 µL of lysate was mixed with 50 µL of CellTiter-Glo reagent in a 96-well plate in triplicate according to the manufacturer’s instructions. Samples were mixed on a horizontal shaker for 2 min at 170 RPM and then incubated in the dark for 10 min before reading. Luminescence was then measured on the BMG fluorostar to determine ATP concentrations which were normalised to protein content.

### Mitochondrial superoxide production

In vivo superoxide production was performed as described previously^46^. Briefly, animals were age synchronised by gravity flotation, and placed onto 3 cm NGM plates as L1 larvae. Both the MitoSOX probe (10 μM final) and sulfur/H_2_S compounds were diluted to a final plate volume of 2.3 mL (2 mL NGM, 200 μL OP50, 100 μL MitoSOX ± compounds). Animals were grown to young adult stages on these plates and then picked over onto seeded OP50 plates for 1 h before imaging to clear residual dye within the gut. Animals were picked into 20 μL of M9 on slides and gently immobilised with a coverslip. Photos were taken using a ×40 objective on an inverted fluorescent microscope with green light excitation, with a set exposure rate of 2 seconds and gain of 3.4 D.b. Images were analysed in ImageJ by manually circling around the terminal bulb of each animal, and measuring the mean integrated density of raw image files. For visual clarity, we used the fire LUT in ImageJ (min/max values kept constant at 0/255, respectively) to display superoxide fluorescence.

### Mitochondrial membrane potential

Membrane potential was measured using a potentiometric fluorescent probe, MitotrackerCMXRos (Invitrogen, UK) that sequesters in mitochondria dependant on proton gradients. Animals were grown to young adult stage with or without respective SAA seeded on plates as described above. A 940 μM stock of the probe was made up in 100% DMSO and diluted to 5 μM (0.5% DMSO) with 40 μL placed into black tubes. Approximately 40 animals were picked into each 40 μL tube and incubated at 20 °C for 1 h in the dark. Before imaging, animals were washed 3× with M9 to remove any residual dye on the outer cuticle of the animal. Animals were then pipetted onto slides and immobilised with a coverslip and imaged in dim lighting. Images were taken using a ×40 objective on an Olympus BX43 fluorescent microscope with green light excitation, with exposure set to 500 ms for all conditions. For quantification, the integrated density was measured and normalised to the area of measurement.

### Antimycin A assay

We performed the Antimycin A exposures as previously described^13^. Briefly, animals were grown to young adult stages as described above. A stock of Antimycin A (Sigma, UK) was made in 100% ethanol and diluted on the day of use to 2 μM in M9 (0.1% ethanol). 1 mL of 2 μM Antimycin A was put into OP50 seeded 3 cm NGM plates, where animals were picked over and incubated for 24 h. For thrashing counts, 15–20 animals were analysed per condition.

### Statistics and reproducibility

Normality was initially assessed using the D’Agostino and Pearson tests, and then the statistical test was selected based on normality. Statistical differences were assessed using either one-way ANOVA, two-way ANOVA, or Kruskal–Wallis test. Significance was determined as *P* < 0.05, and all statistical analyses were completed using GraphPad Prism (USA). Details of numbers of animals used and replicates employed for each analysis are provided in the figure legends for each analysis.

### Reporting summary

Further information on research design is available in the Nature Portfolio Reporting Summary linked to this article.

## Supplementary information

Supplementary Figure 1

Description of Additional Supplementary Files

Supplementary Data 1

Supplementary Data 2

Supplementary Data 3

Supplementary Data 4

Supplementary Data 5

Supplementary Data 6

Supplementary Data 7

Supplementary Data 8

Supplementary Data 9

Supplementary Data 10

Reporting Summary

## Data Availability

The data used for generating the figures are found in Supplementary Datasets 1–10. All supporting data are available upon request from the corresponding author.
