# Supplementary material for: Sulfur amino acid supplementation displays therapeutic potential in a C. elegans model of Duchenne muscular dystrophy
Source: Commun Biol. 2022 Nov 16;5:1255. doi: 10.1038/s42003-022-04212-z (PMC9668843; doi:10.1038/s42003-022-04212-z)
Supplement: Supplementary file 1 — Supplementary Figure 1 [file 42003_2022_4212_MOESM1_ESM.pdf]

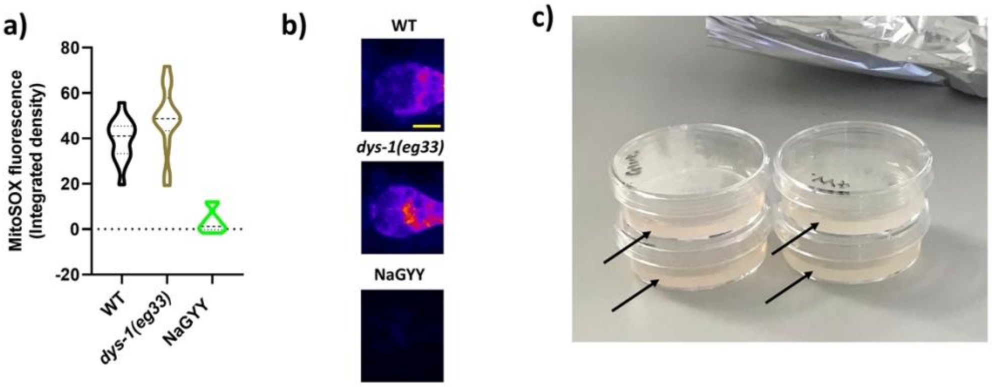

Supplementary Figure 1: When MitoSOX is diluted into plates for worm growth/ incubation (10 µM) for superoxide detection, the plates display a light red/pink colour (see plates on the left in image). This remains consistent with all SAA's and both AP39 concentrations. However, when NaGYY is incorporated into plates (on the right), notice the fluorescence is no longer detected suggesting a direct quenching of the MitoSOX probe. The significantly low superoxide signal is therefore, a false positive from insufficient probe being taken up by animals, an important finding for those performing superoxide measures with NaGYY/ other salt-based H_2S donors. Data are presented as violin plots to display the distribution of the data. Dashed lines represent the median and quartiles are represented by the dotted lines. Scale bar: 20 µm. Data are obtained from two biological repeats with 20-30 animals per condition.
