# Supplementary material for: Sulfur amino acid supplementation displays therapeutic potential in a C. elegans model of Duchenne muscular dystrophy
Source: Commun Biol. 2022 Nov 16;5:1255. doi: 10.1038/s42003-022-04212-z (PMC9668843; doi:10.1038/s42003-022-04212-z)
Supplement: Supplementary file 2 — Description of Additional Supplementary Files [file 42003_2022_4212_MOESM2_ESM.pdf]

Description of Additional Supplementary Files

File name: Supplementary Data 1

Description: Data used for Figures 1 and 2 – H2S levels

File name: Supplementary Data 2

Description: Data used for Figure 1 qPCR

File name: Supplementary Data 3

Description: Data used for Figure 2 swim assays

File name: Supplementary Data 4

Description: Data used for Figure 3 lost nuclei

File name: Supplementary Data 5

Description: Data used for Figure 4 GFP analysis

File name: Supplementary Data 6

Description: Data used for Figure 5 mitochondrial membrane

File name: Supplementary Data 7

Description: Data used for Figure 6 mitoSox

File name: Supplementary Data 8

Description: Data used for Figure 7 Calcium levels

File name: Supplementary Data 9

Description: Data used for Figure 7 Levamisole sensitivity

File name: Supplementary Data 10

Description: Data used for Figure 8
